# The Prevalence of Tb in HIV Patients and Risk Factor With Frequent Referral (Iran, 2009-10)

**DOI:** 10.5812/ircmj.4401

**Published:** 2013-01-05

**Authors:** Narmela Rabirad, Esmaeil Mohammad Nejad, Mohammad Reza Hadizadeh, Jamaloddin Begjan, Seyyedeh Roghayeh Ehsani

**Affiliations:** 1 Department of Infection Control, Tehran University of Medical Sciences, Tehran, IR Iran; 2 Department of Nursing, International Branch, Shahid Beheshti University of Medical Sciences, Tehran, IR Iran; 3 Department of Infectious, Mashhad University of Medical Sciences, Mashhad, IR Iran; 4 Department of Nursing, Faculty of Nursing and Midwifery, Tehran University of Medical Sciences, Tehran, IR Iran; 5 Department of Nursing, Imam Khomeini Clinical and Hospital Complex, Tehran University of Medical Sciences, Tehran, IR Iran

**Keywords:** HIV, Tuberculosis, Prevalence, Coinfection

## Abstract

**Background:**

HIV infection significantly increases the risk of tuberculosis and this disease is one of the most common opportunistic infections in HIV Patients.

**Objectives:**

The aim of this study was to determine of the prevalence of tuberculosis and risk factor among HIV patients.

**Materials and Methods:**

In this cross-sectional study, from September 2009 to September 2010, 71 HIV patients who referred to teaching hospital in Tehran, Capital Iran were enrolled. Records of patients that admitted to hospital at least once a year and have positive test for HIV with ELISA and Western Blot were assessed. TB diagnosis testes included clinical finding, chest X-Ray and culture positive sputum.

**Results:**

74.6 % and 38 % of them had prison history and history of addiction respectively, 28.2% of subjects co- infected with HIV / TB and 40% of patients were under treatment with anti-retroviral drugs. There was relationship Between TB and CD4 counts below 200 cells per micro-liters (P = 0.003), age (P = 0.000), anti-retroviral drugs (P = 0.001), discharge status (P = 0.004), prison history (P = 0.002) and alcohol and smoking (P = 0.01).

**Conclusions:**

The prevalence rate of TB / HIV among intravenous drug abusers and prisoners was high also the prevalence of tuberculosis among HIV patients.

## 1. Introduction

Although Human immunodeficiency virus (HIV) infection is considered pandemic by the World Health Organization(WHO) but is spreading rapidly despite enormous efforts to control its spread (1). Tuberculosis is one of the famous and ancient contagious diseases and from years ago was known as one of the problems of healthcare system and cause of death worldwide especially in third world countries. World statistics shows well the extent and significance of this chronic and debilitating disease that is located the head group of diseases and disorders. In 1393 the World Health Organization introduced the disease a global threat and estimates that in 2005 approximately 33 percent of the world populations are infected with TB (2). Some countries with high HIV prevalence, up to 80% of TB patients have HIV infection (3). More than half of these patients live in Asia and in developing countries has had a fixed status and almost without loss. Iran as a developing country is not safe from harm of this disease and incidence of disease in Iran have been reported about 8 per one hundred thousand people (4). The incidence of tuberculosis in patients with HIV infection in Asian countries where TB is endemic showing the growing sensitivity of AIDS to the TB germs and more than 9 percent of TB cases with HIV occurs in these countries (5). Today, the statistics especially The World Health Organization statistics shows that about 1.2 to 1.3 of 40 million people infected with HIV is suffering from tuberculosis. Approximately 11.8 million people worldwide are infected with both disease, and 2 percent of deaths occur due to co-infection of TB / HIV (6). One death happens every 30 seconds among TB patients in the world for unknown reasons (7). Tuberculosis is the most common cause of death among these patients and one of 3 co-infected patients dies of because TB and mortality risk in such patients is four times of patients with TB without HIV and the disease will become worse if left untreated (8, 9). Due to the worrisome increase in HIV infection, the tuberculosis is increasing also (2). This study was performed to determine the Prevalence of Tb in HIV Patients and Risk Factor with recurrent referral in 2009-10.

## 2. Materials and Methods

This cross - sectional study was conducted based on the review of HIV / AIDS patients documents available in the archives of a teaching hospital in Tehran capital Iran from September 2009 to September 2010. Documents of patients who were admitted at least once and had positive ELISA test and Western Blot for HIV had been accepted and 71 patients met inclusion criteria. The research instrument was a questionnaire that its validity confirmed by 10 faculty members of Tehran University of Medical Sciences and Islamic Azad University of Tehran and its reliability was measured by test re test. The questionnaire contains information such as age, sex, marital status, education level, insurance status, exposure to the virus HIV, the number of referral, year of diagnosis, type of addiction, prison history, alcohol, clinical syndrome led to hospitalization and CD4 count that was completed by visiting patients and their documents during their hospitalization. The clinical syndrome leading to hospitalization is considered based on final diagnosis and the latest CD4 count of patients. TB was diagnosed: based on clinical findings, laboratory and chest X-Ray (according to the physician's records in the patients file), cluture positive sputum and patients were isolated and treated with anti-TB drugs. Questions were completed referring to patient records and interview with them. For data analysis we initially used descriptive statistics including frequency tables, graphs and measures of central value and measures of dispersion to describe the study variables and then using the chi-square test the relationships between variables was evaluated. All the data were analyzed by SPSS version 16 and P < 0/05 was considered significant.

## 3. Results

The mean age of subjects was 35 ± 8.1 which range 17 to 61 years, 37 patients (52.1%) were in the age range 25 to 35 years and 91.5% and 8.1% of them were male and female respectively. The most common way of HIV morbidity was intravenous drug abuse (64.8%) and overally74.6 % and 38 % of patients had a drug injection and a prison history respectively (Table 1).

**Table 1:** Demographic Data of HIV Patient With Recurrent Referral in 2009-10

Variable	HIV positive, No (%)	TB positive, No (%)
**sex**		
male	65 (91.5)	20 (100)
female	6 (8.5)	0 (0)
**Age(year)**		
Less than 24 Y/O	6 (8.5)	2 (10)
24.1 - 34	38 (53.5)	13 (65)
34.1 – 44	23 (32.4)	6 (30)
More than 44.1 Y/O	4 (5.6)	0 (0)
**Educational level**		
Less than high school education	58 (81.7)	14 (70)
Diploma and diploma of associate degree	12 (16.9)	6 (30)
Higher education degree	1 (1.4)	0 (0)
**Marital status**		
Single	23 (32.4)	6 (30)
Married	38 (53.5)	11 (55)
Divorced	10 (41.1)	3 (5)
**Insurance status**		
Social (public) insu.	3 (4.2)	2 (10)
Health care insu.	26 (36.6)	5 (25)
Other insu.	5 (7.04)	2 (10)
No insu.	37 (52.1)	11 (55)
**Ways of AIDS morbidity(transmission)**		
Shared intravenous needles	46 (64.8)	14 (70)
Sexually transmission	20 (28.1)	5 (25)
Septic blood products	2 (2.8)	0 (0)
Other ways	3 (4.2)	1 (5)
**Prison history**				
Yes	27 (38)	10 (50)
No	44 (62)	10 (50)
**Addiction history**		
Yes	53 (74.6)	17 (85)
No	18 (25.4)	3 (15)
**Alcohol**		
Yes	48 (67.6)	8 (40)
No	23 (32.4)	12 (60)
**Cigarette**		
Yes	54 (76.05)	18 (90)
** No**	17 (23.94)	2 (10)

Of 71 HIV infected patients, 28.2 percent were diagnosed with pulmonary tuberculosis, had the mean age of 31 ± 4.3 which range 18 to 43 years and their mean CD4 count was 143.95 cells per ml which range 26 to 506 cells per ml. All patients with tuberculosis were males and 50%, 85%, 40% and 90% of them had history of prison, intravenous drug abuse, alcohol and cigarette smoking respectively. 74% of patients had CD4 less than 200 ml per m3. 40% of patients co-infected with HIV/TB were treated with anti-retroviral drugs. 45% and 35% of patients were diagnosed with HIV infection in 2010 and 2009 respectively and 10% of patients died during the hospitalization. The patients’ average hospital stay in the first referral was 14.6 days which range 2 to 43 days but the average hospital stay in the last referral was 12.5 days which range 2 to 40 years. Other demographic data is shown in Table 1. Our finding showed that between tuberculosis and treatment with anti-viral drugs (P = 0.001), prison history (P = 0.002), discharge status (P = 0.04), smoking and alcohol history (P = 0.01) there was a relationship but there was no significant relationship between the educational level, way of AIDS morbidity and year of diagnosis with pulmonary tuberculosis.

## 4. Discussion

Pulmonary tuberculosis is one of most common infection among HIV/ AIDS patients so that World Health Organization in 2005 estimates that 33 percent of people in Asia are co- infected with the TB / AIDS (10). In our study, the prevalence of pulmonary tuberculosis in patients infected with HIV was 28.2%. In Iran , similar to our result , Mohraz et al. and Mohammadnejad et al. showed that prevalence were14.7% and12.7%, respectively (11, 12). In our study, 8.5% of the hospitalized patients were women and 91.5 % were male.74.6% and 35.5% of patients had a history of addiction and prison respectively. According to The Iranian Center of Disease Management 95.5% of HIV / AIDS cases are men and only 4.5 % are women. Also 66.4% of cases of HIV infection transmitted through injecting drug abuse and 12.6 % from other ways and in 21% of cases way of transmission was unknown. The important point in this study is that unknown cases in The Iranian Center of Disease Management can be in the sexually transmission phase, although based on the World Health Organization during1996-2003 a significant increase in the prevalence of sexually transmitted disease that cause ulcers was reported (13).

Results shown that 40% of co-infected patients with HIV / TB were in the age range 21-30 years and 30% were in the age range 31-40 years and the mean age of coinfected patients with HIV / TB was lower than other patients who were not coinfected (14). According to The World Health Organization reports in 2005, retroviral treatment coverage in The Middle East is estimated about 5% and 10% in Iran (15). Findings showed that 75 percent of HIV / TB patients have had CD4 less than 200 ml, but in a study on 20 patients with TB / HIV and 73 patients without TB, showed that CD4 count was less than 200/mm3 (16). Prevalence of HIV/ TB in injecting drug abusers and prisoners is high; therefore it is require a full medical examination in prison for injecting drug abusers to prevent transmission of infectious diseases like tuberculosis.
